# SMOTE-DRNN: A Deep Learning Algorithm for Botnet Detection in the Internet-of-Things Networks

**DOI:** 10.3390/s21092985

**Published:** 2021-04-24

**Authors:** Segun I. Popoola, Bamidele Adebisi, Ruth Ande, Mohammad Hammoudeh, Kelvin Anoh, Aderemi A. Atayero

**Affiliations:** 1 Department of Engineering, Manchester Metropolitan University, Manchester M1 5GD, UK; segun.i.popoola@stu.mmu.ac.uk (S.I.P.); ruth.e.ande@stu.mmu.ac.uk (R.A.); 2 Department of Computing and Mathematics, Manchester Metropolitan University, Manchester M1 5GD, UK; m.hammoudeh@mmu.ac.uk; 3 School of Engineering, University of Bolton, Greater Manchester BL3 5AB, UK; k.anoh@bolton.ac.uk; 4 Department of Electrical and Information Engineering, Covenant University, Ota P.M.B. 1023, Nigeria; atayero@covenantuniversity.edu.ng

**Keywords:** botnet, cybersecurity, deep learning, intrusion detection, Internet of Things

## Abstract

Nowadays, hackers take illegal advantage of distributed resources in a network of computing devices (i.e., botnet) to launch cyberattacks against the Internet of Things (IoT). Recently, diverse Machine Learning (ML) and Deep Learning (DL) methods were proposed to detect botnet attacks in IoT networks. However, highly imbalanced network traffic data in the training set often degrade the classification performance of state-of-the-art ML and DL models, especially in classes with relatively few samples. In this paper, we propose an efficient DL-based botnet attack detection algorithm that can handle highly imbalanced network traffic data. Specifically, Synthetic Minority Oversampling Technique (SMOTE) generates additional minority samples to achieve class balance, while Deep Recurrent Neural Network (DRNN) learns hierarchical feature representations from the balanced network traffic data to perform discriminative classification. We develop DRNN and SMOTE-DRNN models with the Bot-IoT dataset, and the simulation results show that high-class imbalance in the training data adversely affects the precision, recall, F1 score, area under the receiver operating characteristic curve (AUC), geometric mean (GM) and Matthews correlation coefficient (MCC) of the DRNN model. On the other hand, the SMOTE-DRNN model achieved better classification performance with 99.50% precision, 99.75% recall, 99.62% F1 score, 99.87% AUC, 99.74% GM and 99.62% MCC. Additionally, the SMOTE-DRNN model outperformed state-of-the-art ML and DL models.

## 1. Introduction

The Internet-of-Things (IoT) paradigm enables physical objects to interconnect and communicate with each other via the Internet [1]. The popularity of IoT is fast-growing, and its adoption cuts across different areas of application such as energy, water, transport, defense, health, agriculture, etc. According to Cisco’s Annual Internet Report, 14.7 billion IoT devices will be connected to the Internet by 2023 [2].

On the other hand, botnet is a network of compromised computers, known as bots, that are remotely controlled by a botmaster using a Command and Control (C&C) server [3]. Nowadays, hackers leverage botnets, such as *Mirai* [4,5], to exploit vulnerabilities in IoT devices and networks. They use this technique to launch different types of cyber-attacks against Internet-enabled infrastructures [6,7,8,9,10,11]. For example, in September 2016, the *Mirai* botnet compromised several IoT devices to launch a Distributed Denial of Service (DDoS) attack, increasing traffic up to 620 Gbps in a web server [5]. Additionally, in October 2016, a web-host and cloud service provider, *Dyn*, was hit with a DDoS attack, increasing traffic to about 1.1 Tbps. The *Mozi* botnet was first discovered in October 2019. It accounted for close to 90% of the total IoT network traffic monitored by IBM Security X-Force from that time until June 2020; this incidence increased IoT attack volume by 400% compared to the total IoT attack cases in the last two years [12].

Cybercriminals can manipulate the energy market for a significant payoff of up to $24 million if they have access to 50,000 high-wattage IoT devices for only three hours a day, 100 days a year [13,14]. Additionally, with the current COVID-19 pandemic, corporate and IoT networks have become more vulnerable to botnet attacks because these networks are now being accessed remotely more often than before [15]. Recent and complex botnet attacks that target IoT networks include Denial of Service (DoS), DDoS, Operating System (OS) Fingerprinting (OSF), Service Scanning (SS), Data Ex-filtration (DE) and Keylogging (KL) [16]. A unique DoS or DDoS attack can be launched using either a Hypertext Transfer Protocol (HTTP), a Transmission Control Protocol (TCP) or a User Datagram Protocol (UDP). Such botnet attacks are referred to as DoS-HTTP, DoS-TCP, DDoS-UDP, DDoS-HTTP, DDoS-TCP or DDoS-UDP attacks.

Botnet attack detection in IoT networks can be formulated as a classification problem [16]. For binary classification, each sample in a network traffic packet is classified as either benign or malicious based on certain predefined features. On the other hand, the specific category of botnet attack is identified in multi-class classification. Thus far, Artificial Intelligence (AI) techniques have achieved good performance in handling classification tasks in different application areas including voltage stability assessment of power systems among many others [17]. Specifically, various Machine Learning (ML) and Deep Learning (DL) models have been developed to classify network traffic data in IoT networks. These models learn the discriminating features of benign traffic and malicious traffic using different architectures such as Random Forest (RF) [18], Support Vector Machine (SVM) [19], Deep Neural Network (DNN) [20], Recurrent Neural Network (RNN) [21], Long Short-Term Memory (LSTM) [22] and Gated Recurrent Unit (GRU) [23]. For an in-depth understanding, comprehensive reviews and surveys on the application of ML and DL in intrusion detection are presented in [24,25,26,27,28,29].

Classification of highly imbalanced network traffic data is a difficult task. The data are said to be highly imbalanced when the ratio of the number of samples in the majority class to that of the minority class is more than 1:10 [30]. High class imbalance degrades the classification performance of ML and DL models in minority classes [31,32]. In addition to the class imbalance problem, small disjuncts, noise and overlap in network traffic samples can also lead to poor classification performance [33,34]. In popular botnet attack scenarios such as DDoS, the amount of malicious traffic generated is usually far more than the volume of benign traffic produced by legitimate devices in an IoT network. This means that the number of samples in the *normal* class is very low compared to the number of samples in the *attack* class. In this case, state-of-the-art DL models tend to be biased in favour of the majority (*attack*) class, and this increases the false positive (FP) rate [16]. The implication of deploying state-of-the-art ML and DL models in real-life IoT networks is that a significant percentage of network traffic data in the minority classes is misclassified, and this may, consequently, lead to a breach of privacy, loss of sensitive information, loss of revenue when applications and services are unavailable, and even loss of lives in critical IoT systems.

In this paper, we propose an efficient DL-based botnet attack detection algorithm to increase the detection rate and to reduce FP in minority classes without increasing the false negative (FN) rate in the majority classes. The main contributions of this paper are as follow:

1. An efficient DL-based botnet attack detection algorithm is proposed for highly imbalanced network traffic data. Synthetic Minority Oversampling Technique (SMOTE) generates additional minority samples to achieve class balance, while Deep RNN (DRNN) learns hierarchical feature representations from the balanced network traffic data to perform discriminative classification.
2. DRNN and SMOTE-DRNN models are trained, validated and tested with the Bot-IoT dataset to classify network traffic samples in the *normal* class and ten botnet attack classes.
3. We investigate the effect of class imbalance on the accuracy, precision, recall, F1 score, false positive rate (FPR), negative predictive value (NPV), area under the receiver operating characteristic curve (AUC), geometric mean (GM) and Matthews correlation coefficient (MCC) of the DRNN and SMOTE-DRNN models.
4. The training time and the testing time of the DRNN and SMOTE-DRNN models are analysed to evaluate their training speed and detection speed.

The rest of the paper is organised as follows: In Section 2, we review the state-of-the-art ML and DL methods proposed for botnet attack detection in IoT networks; in Section 3, we present a detailed description of the SMOTE-DRNN algorithm and model development process; in Section 4, we discuss the results; and in Section 5, we summarise the main findings of the paper.

## 2. Review of Related Works

In this section, we review the state-of-the-art ML and DL models that were developed with the Bot-IoT dataset [16] to detect botnet attacks in IoT networks. Currently, the Bot-IoT dataset is the most relevant and up-to-date publicly available dataset for botnet attack detection in IoT networks because it (a) has IoT network traffic samples, (b) captures complete network information, (c) has a diversity of complex IoT botnet attack scenarios, (d) contains accurate ground truth labels and (e) provides a massive volume of labeled data required for effective supervised DL.

In recent literature, researchers have recommended diverse ML and DL model architectures for botnet detection in IoT networks. Odusami et al. [35] proposed LSTM for DDoS attack detection in web servers, but they did not perform any experiment to validate the performance of the proposed method. Biswas and Roy [36] proposed the GRU model because it outperformed the Artificial Neural Network (ANN) and LSTM models. However, the performance evaluation was based on accuracy only. Popoola et al. [21] proposed Stacked RNN (SRNN), which involves cascading multiple layers of RNN. Tyagi and Kumar [37] recommended the RF model because it performed better than the k-Nearest Neighbour (kNN), Logistic Regression (LR), SVM, Multi-Layer Perceptron (MLP) and Decision Tree (DT) models. Lo et al. [38] proposed the Edge-based Graph Sample and Aggregate (E-GraphSAGE) model and it outperformed the Extreme Gradient Boosting (XGBoost) and DT models. Chauhan and Atulkar [39] suggested the Light Gradient Boosting Machine (LGBM) model because it outperformed RF, Extra Tree (ET), Gradient Boost (GB) and XGBoost models. In [40], the Convolutional Neural Network (CNN) model outperformed the RNN, LSTM and GRU models. Huong et al. [41,42] proposed a low-complexity edge-cloud DNN model, which achieved better performance than the kNN, DT, RF and SVM models. Lee et al. [43] employed RF for botnet attack classification in an IoT smart factory. Shafiq et al. [44] developed the Bayes Network (BN), C4.5 DT, Naive Bayes (NB), RF and Random Tree (RT) models. The bijective soft set algorithm [45] was used to determine the most effective ML model based on accuracy, precision, recall and training time. Zakariyya et al. [46] recommended LGBM as a resource-efficient ML method. Susilo et al. [47] proposed the CNN model for botnet detection in Software-Defined Networks (SDN), and it outperformed the RF model. In [48], the DNN model achieved a higher classification accuracy than the LR, kNN, DT, Classification And Regression Tree (CART) and SVM models. Das et al. [49] developed the RF, RT, NB, C4.5 DT, Reduced Error Pruning Tree (REPT), BN and partial decision tree (PART) models with the 10 best features in [16]. In [50], the DT model outperformed the NB, kNN and SVM models. Sriram et al. [51] concluded that DL models perform better than classical ML models.

In another group of studies, two or more ML/DL architectures were combined to form a hybrid model. Popoola et al. [22] proposed a hybrid DL method based on the combination of LSTM Autoencoder (LAE) and deep Bidirectional LSTM (BLSTM) model architectures. LAE reduces the dimensionality of network traffic features to save memory space and to increase computation speed. On the other hand, deep BLSTM learns the long-term temporal relationships among the low-dimensional features to correctly distinguish between benign traffic and different classes of botnet attack traffic. Priya et al. [52] combined SVM, NB, DT, RF and ANN to develop an ensemble classifier for attack detection in Industrial IoT (IIoT). Kunang et al. [53] proposed a hybrid DL method by cascading Autoencoder (AE) and DNN model structures. AE performs feature extraction, while DNN performs the classification task. Zixu et al. [54] merged Generative Adversarial Network (GAN) with AE to detect botnet attacks in distributed IoT networks. Ge et al. [55] combined the concept of transfer learning with DNN. Bhuvaneswari and Selvakumar [56] developed the Vector Convolutional Deep Learning (VCDL) model, which employed a vector convolutional network for feature extraction and a fully connected network for classification. Asadi et al. [57] developed a hybrid ML model, which comprised the DNN, SVM and C4.5 decision tree algorithms. Khraisat et al. [58] developed a hybrid ML model, which comprised One-Class SVM (OCSVM) and C4.5 DT. Aldhaheri et al. [59] developed a hybrid between the Self Normalising Neural Network (SNN) and Dendritic Cell Algorithm (DCA) models with five optimal network traffic features, which were selected from the 10 best features in [16] using the information gain method [60].

Furthermore, different optimisation techniques were proposed to improve the classification performance of ML and DL models. Popoola et al. [23] proposed a method that helps determine the most appropriate set of hyperparameters for training the Bidirectional GRU (BGRU) model in an efficient manner. Samdekar et al. [61] recommended the Firefly Algorithm (FA) for feature dimensionality reduction because it outperformed the Chi-Square, ET and Principal Component Analysis (PCA) methods when SVM was used for classification. Kumar et al. [62] proposed the combination of the correlation coefficient, RF mean decrease accuracy and gain ratio for selection of the most relevant features. Kunang et al. [53] and Injadat et al. [63] proposed the Bayesian Optimisation Gaussian Process (BO-GP) method to optimise the hyperparameters of the AE-DNN and DT models, respectively. In [64], Binary Grey Wolf Optimisation (BGWO) was used for feature selection while NB was used for classification. Orevski and Androcec [65] used Genetic Algorithm (GA) to optimise the hyperparameters of ANN. In [66], Particle Swarm Optimisation (PSO) algorithm was used to determine the best hyperparameters that maximise AUC.

In the literature, botnet attack detection in IoT networks was treated as a classification task. Different ML and DL models have been developed for botnet attack detection in binary, 5-class or 11-class classification scenarios. For binary classification, ML/DL models were developed such that each sample of the network traffic data in the Bot-IoT dataset was classified as either *normal* or *attack*. For 5-class classification, four categories of botnet attacks, namely DDoS, DoS, reconnaissance and theft, were considered. For the 11-class classification scenario, ten categories of botnet attacks, namely DDoS-HTTP (DDH), DDoS-TCP (DDT), DDoS-UDP (DDU), DoS-HTTP (DH), DoS-TCP (DT), DoS-UDP (DU), OS fingerprinting (OSF), service scanning (SS), data exfiltration (DE) and keylogging (KL), were considered. In order to cover all of the available botnet attack types, our study focuses on botnet attack detection in an 11-class classification scenario.

Ferrag et al. [67] proposed an RNN model that employs a single hidden layer and 60 hidden neurons. The model was trained with 1,878,561 network traffic samples for five epochs using a batch size of 100. The classification performance of the RNN model was evaluated with 1,797,803 network traffic samples in the test set. The RNN model outperformed the SVM, RF and NB models. The RNN model had the highest recall value for each of the 10 botnet attack classes. Furthermore, the overall recall and the FPR of the RNN model were the highest and lowest, respectively.

Ferrag et al. [68] investigated the effectiveness of three deep discriminative models (DNN, RNN and CNN) and four deep generative models (Restricted Boltzmann Machine (RBM), Deep Belief Network (DBN), Deep Boltzmann Machine (DBM) and Deep AE (DAE)) for botnet attack detection in IoT networks. These models employed a single hidden layer and 15–100 hidden neurons. The activation functions at the hidden layer and the output layer were sigmoid and softmax, respectively. The DL models were trained with 5,877,647 network traffic samples for 100 epochs, given a learning rate of 0.01–0.5 and a batch size of 1000. The classification performance of the DL models was evaluated with 1,469,413 network traffic samples in the test set. The authors presented the recall of the DL models for the 10 botnet attack classes. Additionally, the overall recall, TNR, FPR and accuracy were reported. The CNN model outperformed the DNN and RNN models, while the DAE model outperformed the RBM, DBN and DBM models.

Ferrag et al. [69] combined the REP Tree, JRip and Forest PA algorithms to form a hybrid ML model named RDTIDS. The REP Tree and JRip models were trained with 5,877,647 network traffic samples to perform binary classification. The outputs of the two models were combined with the network traffic features in the training set to develop the Forest PA model for multi-class classification. The classification performance of the RDTIDS model was evaluated with 1,469,413 network traffic samples in the test set. The authors presented the recall of the RDTIDS model for the 10 botnet attack classes. Additionally, the overall recall, FPR and accuracy were reported. The RDTIDS model outperformed the RF, REP Tree, MLP, NB, JRip, SVM and J48 models.

Alkadi et al. [70] proposed a BLSTM model that employed a single hidden layer and 60 hidden neurons. BLSTM model was trained, validated and tested with 60%, 20% and 20% of the network traffic samples for 200 epochs, given a batch size of 100 and an Adam optimiser. The activation functions at the hidden layer and the output layer were hyperbolic tangent (*tanh*) and softmax, respectively. The authors presented the recall of the BLSTM model for the 10 botnet attack classes. Additionally, the overall recall, FPR and accuracy were reported. The BLSTM model outperformed the SVM, RF and NB models.

SMOTE is a method that can effectively handle the class imbalance problem in training data, but it must be combined with the right classifier. Pokhrel et al. [71] proposed SMOTE-kNN to address the class imbalance problem in botnet detection. However, the study focused on binary classification, and the IR in the training data was 1:208. Bagui and Li [72] studied the effects of random undersampling (RU), random oversampling (RO), RU-RO, RU-SMOTE and RU with Adaptive Synthetic (ADSYN) methods on the performance of the ANN model. Qaddoura et al. [73] combined SVM-SMOTE with DNN to handle a class imbalance in binary classification. Derhab et al. [74] employed a combination of SMOTE and Temporal CNN to address the class imbalance in 5-class classification. However, the SMOTE method has not been previously combined with the DRNN model. Additionally, previous applications of SMOTE focused on binary and 5-class classification, but none of them applied it to solve the 11-class classification problem.

Table 1 shows that the distribution of network traffic samples in the training set is highly imbalanced across the 11 classes. The number of samples in the minority classes (DDH, DH, Norm, DE and KL) is relatively fewer than those in the majority classes (DDT, DDU, DT, DU, OF and SS). For majority classes, high class imbalance in the training set degrades the classification performance of state-of-the-art ML and DL models. Therefore, state-of-the-art ML and DL models may not detect DDH, DH, Norm, DE and KL correctly in IoT networks. In this paper, the number of samples in the Norm, DE and KL classes is relatively few compared to those in the previous studies. Therefore, the class imbalance problem in the present study is more challenging. Additionally, in previous related work [67,68,69,70], the recall values for the Norm class were not reported and the authors did not present the accuracy, precision, F1 score, FPR, NPV, AUC, GM and MCC of the ML/DL models for each of the 11 classes.

## 3. SMOTE-DRNN Algorithm and Model Development

**Figure 1:**
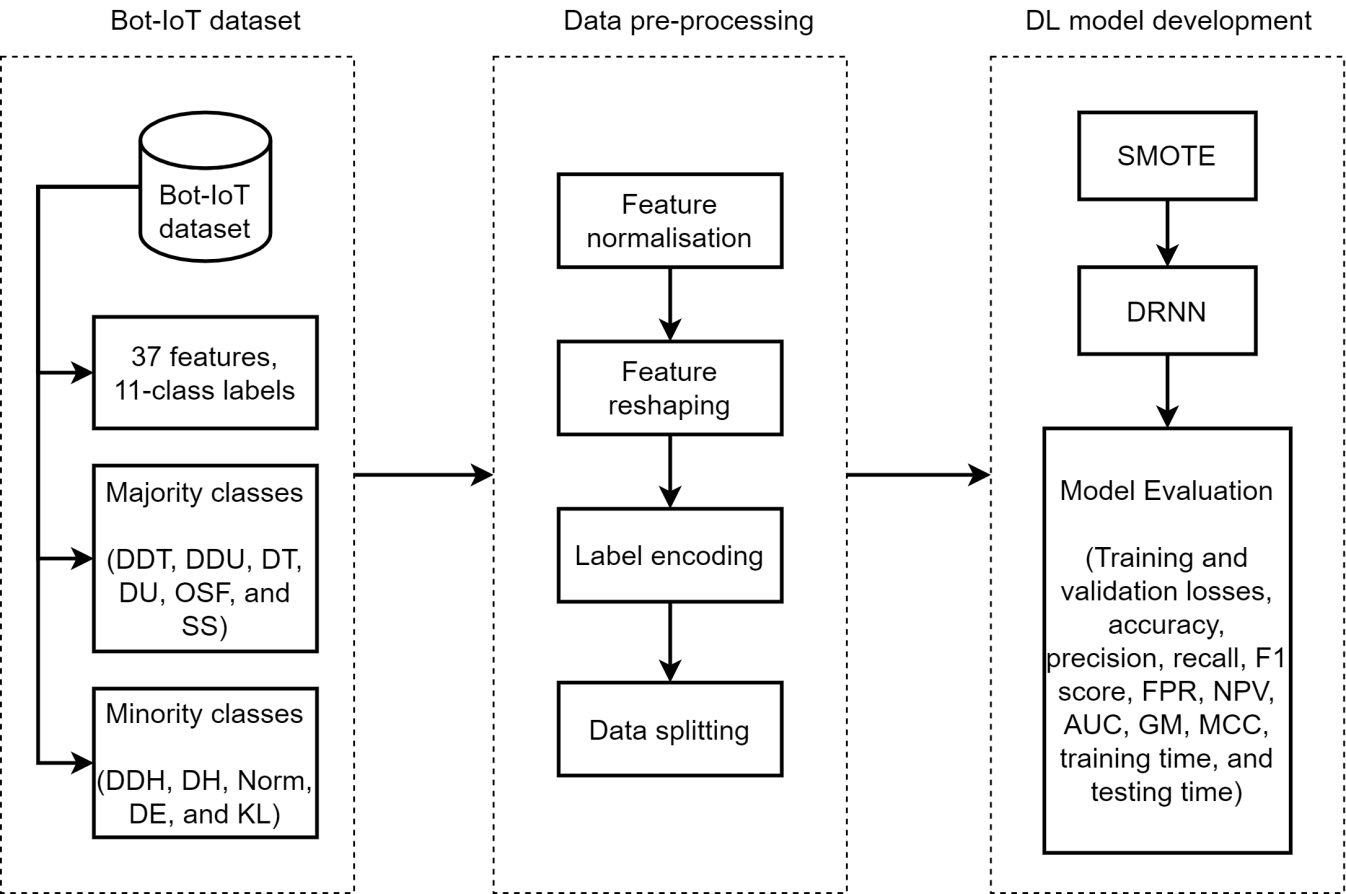
SMOTE-DRNN for botnet attack detection in IoT networks.

In this section, we present the SMOTE-DRNN algorithm and we describe the DL model development process shown in Figure 1. This includes information about the network traffic data, data pre-processing, SMOTE and DRNN.

### 3.1. Network Traffic Data

The Bot-IoT dataset contains 43 network traffic features and three categories of labels for binary, 5-class and 11-class classification each. The names and descriptions of the features and labels are provided in [16]. However, only 37 out of the 43 features were found to be relevant for botnet attack detection in IoT networks. Specifically, *pkSeqID*, *saddr*, *daddr*, *proto*, *state* and *flgs* were excluded. We observed that *pkSeqID*, *saddr* and *daddr* are device-specific, while *proto*, *state* and *flgs* give the same information as *proto_number*, *state_number* and *flgs_number*. In order to detect botnet attacks in a more specific manner, we considered an 11-class classification scenario.

Table 2 shows the sample distribution across the 11 classes in the training, validation and test sets. We noticed that there is high class imbalance in the network traffic data. This often degrades the classification performance of ML and DL models in the minority classes [31,32]. In this paper, the minority classes include DDH, DH, Norm, DE and KE because they have relatively few samples compared to the majority classes, i.e., DDT, DDU, DT, DU, OSF and SS.

### 3.2. Data Preprocessing

The network traffic features and 11-class labels in the training, validation and test sets were transformed into different forms for ease of computation and to achieve better convergence during the DL model development process. The data preprocessing includes feature normalisation, feature reshaping, label encoding and data splitting. First, each of the values of the network traffic features was scaled to a range of 0 and 1 using the min–max normalisation method given by Equation (1) [75,76]:

(1) $$x_{norm}=\frac{x-x_{min}}{x_{max}-x_{min}},$$

where **x** is a network traffic feature vector while $x_{min}$ and $x_{max}$ are the minimum and maximum values of x, respectively. In order to enable development of the DRNN model, an extra dimension was included in the feature set to represent a unit time step (i.e., t = 1). This changed the dimension of the feature set from $X\in R^{p\times q}$ to $X\in R^{p\times 1\times q}$, where *p* is the total number of samples and *q* is the total number of features. Numeric values of the 11 classes in the label vector were represented by integers 0–10. Lastly, as shown in Table 2, the complete network traffic data were randomly split into training (60%), validation (20%) and test sets (20%) to train and evaluate the robustness of DL models against underfitting and overfitting. In this paper, we employed the hold-out validation method to handle overfitting in the neural networks. During model training, the samples in the validation set were used to tune the model hyperparameters. In addition, the generalisation ability of DL models was evaluated with the previously unseen network traffic samples in the test set.

### 3.3. Synthetic Minority Oversampling Technique

The SMOTE algorithm was proposed to deal with the high class imbalance problem in the training set in an 11-class classification scenario. Unlike the method in [77,78], which oversamples minority classes with replacement, the method employed in this paper generates synthetic examples using techniques such as rotation and skew in order to achieve class balance [79].

These synthetic network traffic data were generated along the line segments joining the *k* nearest neighbours of the minority classes, where *k* =3. Therefore, the neighbours from the three nearest neighbours were randomly selected. The step-wise process of SMOTE is presented in Algorithm 1. The generation of synthetic samples (*S*) in the minority classes depends on the number of minority class samples (*T*), the oversampling rate (N%) and the number of nearest neighbours (*k*). If *N* is less than 100%, the minority class samples are randomised. We compute *k* nearest neighbours for each of the minority classes only. This is a function of *N*, the current minority class sample (*i*), the integral multiples of 100 in *N* (*j*) and an array of random numbers (nn_array). Z is an array of original minority class samples, *r* is the number of synthetic samples generated and V is an array of synthetic samples.
**Algorithm 1:** SMOTE algorithm.
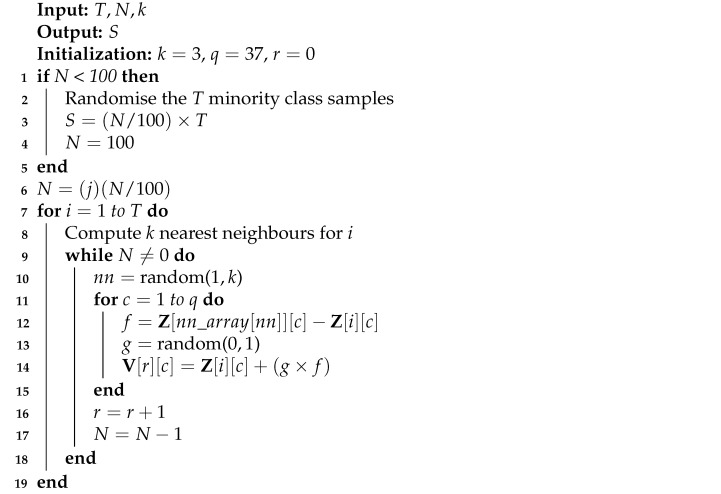


### 3.4. Deep Recurrent Neural Network

The highly imbalanced network traffic data and its corresponding ground-truth labels are represented by $X\in R^{p\times 1\times q}$ and $y\in R^{p\times 1}$, respectively. The DRNN model was trained with $X_{tr}$ and $y_{tr}$ such that the developed classification models can accurately predict $y_{te}$ when only $X_{te}$ is provided in real-life applications. $X_{tr}$ and $X_{te}$ represent known and previously unknown highly imbalanced network traffic data, respectively, while $y_{tr}$ and $y_{te}$ are ground-truth labels of $X_{tr}$ and $X_{te}$, respectively. During model training, the performance of DRNN was confirmed with a different highly imbalanced network traffic data, $X_{va}$, and its ground-truth labels, $y_{va}$. The DL model has a single recurrent layer, four dense layers and a dense output layer.

Unlike Feedforward Neural Network (FNN), RNN has a hidden state that helps model temporal dynamics of input data. RNN learns the temporal dynamics of a mini-batch of highly imbalanced network traffic features, $X_{k}$, by transforming the input data and initial hidden state, $h_{init}$, with trainable parameters, as stated in Equation (2):

(2) $$h_{1k}=\sigma_{h}\left(W_{x}X_{k}+W_{h}h_{init}+b_{h}\right),$$

where $h_{1k}$ is the new hidden state when RNN is trained with the kth mini-batch; $W_{x}$ and $W_{h}$ are the weights used for linear transformation of $X_{k}$ and $h_{init}$, respectively; and $b_{h}$ is the bias. The RNN layer output is further processed based on Equations (3)–(9) to produce a DRNN layer output. Complete information about DRNN is presented in Algorithm 2.

The hidden states of the four dense hidden layers are obtained by Equation (3):

(3) $$h_{mk}=\sigma_{h}\left(W_{hm}h_{(m-1)k}+b_{mh}\right)$$

where m=[2,3,4,5]; $h_{mk}$ is the hidden state of the mth hidden layer; $h_{1k}=h_{k}$; $W_{hm}$ is the weight used for linear transformation of previous hidden state, $h_{(m-1)k}$; $b_{mh}$ is the bias of the mth hidden layer; and $\sigma_{h}$ is a Rectified Linear Unit (ReLU) activation function given by Equation (4):

(4) $$\sigma_{h}\left(a\right)=max\left(0,a\right).$$

If *a* is a negative value, the function returns 0; however, the same *a* is returned when it is a positive value.
**Algorithm 2:** DRNN algorithm.
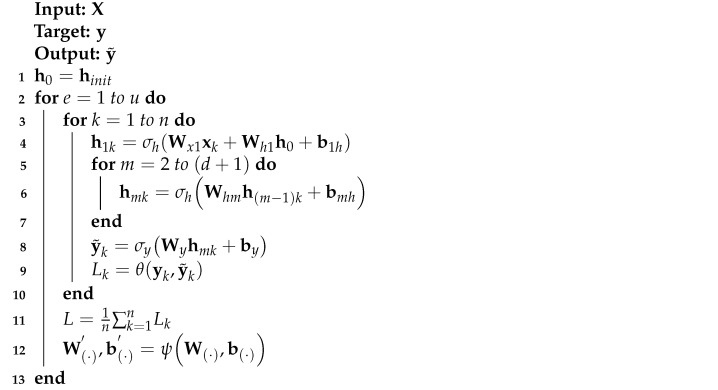


The hidden state of the fourth dense layer, $h_{5k}$, is transformed by the dense output layer, as stated in Equation (5):

(5) $${\tilde{y}}_{k}=\sigma_{y}\left(W_{y}h_{5n}+b_{y}\right),$$

where *n* is the sample size of mini-batch of X and n=p/μ; μ is the batch size, and μ=512; $W_{y}$ is the weight used for linear transformation of $h_{5k}$; $b_{y}$ is the bias of dense output layer; and $\sigma_{y}$ is the activation function of dense output layer.

In the multi-class classification scenario, $\sigma_{y}$ is a softmax function given by Equation (6):

(6) $${\tilde{y}}_{k}=\frac{e^{\left(W_{y}h_{5k}+b_{y}\right)}}{\sum_{\omega =1}^{\gamma }e^{\left(W_{y}h_{5k}+b_{y}\right)}},$$

where γ is the number of classes in y while the difference between $\tilde{y}$ and y is measured by the categorical cross-entropy loss function $\left(\theta_{c}\right)$ in Equation (7):

(7) $$L=\theta_{c}\left(y_{k},{\tilde{y}}_{k}\right)=-\frac{1}{n}\sum_{\tau =1}^{n}\sum_{\omega =1}^{\gamma }\left[y_{\tau ,\omega }\log\left({\tilde{y}}_{\tau ,\omega }\right)\right].$$

The performance of DRNN was validated with different previously unknown highly imbalanced network traffic data, $X_{va}$, and its corresponding ground-truth labels, $y_{va}$. Training loss and validation loss are minimised in mini-batches over *u* epochs using an efficient first-order stochastic gradient descent algorithm named Adam from [80]. The trainable parameters of the densely connected DL model are represented by Equation (8):

(8) $$\Phi =\left[W_{\left(\cdot \right)},h_{\left(\cdot \right)},b_{\left(\cdot \right)}\right].$$

For each epoch, the Adam optimiser, ψ, updates Φ to minimise *L*, as stated in Equation (9):

(9) $$\Phi^{{}^{\prime }}=\psi \left(\Phi ,L,\alpha ,\beta_{1},\beta_{2}\right),$$

where $\Phi^{{}^{\prime }}$ is the new set of trainable parameters, α is the learning rate (0.0001), and $\beta_{1}$ and $\beta_{2}$ are the exponential decay rates (0.9 and 0.999, respectively).

The DRNN and SMOTE-DRNN models were trained with network traffic features and ground truth labels in the Bot-IoT dataset to perform 11-class classification. The performance of ML and DL models depends on the quality of training data, choice of model architecture and selection of appropriate hyperparameters. The right set of model hyperparameters is often determined by extensive experimentation. We adopted the method proposed in [23], and the following hyperparameters were found to be most suitable for our classification task: 100 units each in the RNN and the four dense layers of the DL models, a batch size of 64 and 10 epochs. In this paper, all of the experiments were performed using the Numpy, Pandas, Scikit-learn and Keras libraries that were developed using Python programming language. The Python codes were written and implemented within Spyder Integrated Development Environment (IDE) running on an Ubuntu 16.04 LTS workstation.

## 4. Results and Discussion

In this section, we evaluate the effectiveness of the DRNN and SMOTE-DRNN models with respect to their robustness against underfitting and overfitting as well as their generalisation ability.

### 4.1. Classification Performance Metrics

We compared the classification performance of thet SMOTE-DRNN model with that of the DRNN model and the state-of-the-art ML/DL models based on training loss, validation loss, accuracy, precision, recall, F1 score, FPR, NPV, area under the receiver operating characteristic curve (AUC), geometric mean (GM), Matthews correlation coefficient (MCC), training time and testing time. One of the objectives of this paper is to investigate the effect of highly imbalanced network traffic data on the performance of the DRNN and SMOTE-DRNN models. Therefore, we decided to conduct an extensive investigation that includes all of the performance metrics that are relevant to a typical classification task. Accuracy, precision, recall, F1 score, FPR, NPV, AUC, GM and MCC are represented by Equations (10)–(18):

(10) $$Accuracy=\frac{TP+TN}{TP+TN+FP+FN}\times 100\%,$$

(11) $$Precision=\frac{TP}{TP+FP}\times 100\%,$$

(12) $$Recall=\frac{TP}{TP+FN}\times 100\%,$$

(13) $$F1=\frac{2\times TP}{(2\times TP)+FP+FN}\times 100\%,$$

(14) $$FPR=\frac{FP}{FP+TN}\times 100\%,$$

(15) $$NPV=\frac{TN}{TN+FN}\times 100\%,$$

(16) $$AUC=\frac{1}{2}\left[\frac{TP}{TP+FN}+\frac{TN}{TN+FP}\right]\times 100\%,$$

(17) $$GM=\sqrt{\frac{TP}{TP+FP}\times \frac{TN}{TN+FN}}\times 100\%,$$

(18) $$MCC=\frac{\left(TP\times TN)-(FP\times FN\right)}{\sqrt{\left(TP+FP)(TP+FN)(TN+FP)(TN+FN\right)}}\times 100\%,$$

where TP is the number of IoT botnet attack samples that are correctly classified as IoT botnet attack traffic, FP is the number of normal samples that are misclassified as IoT botnet attack traffic, TN is the number of normal samples that are correctly classified as normal traffic and FN is the number of IoT botnet attack samples that are misclassified as normal traffic.

### 4.2. Robustness against Model Underfitting and Overfitting

In this subsection, we analyse the cross-entropy losses of the DRNN and SMOTE-DRNN models during training and validation to evaluate their robustness against underfitting and overfitting, respectively.

**Figure 2:**
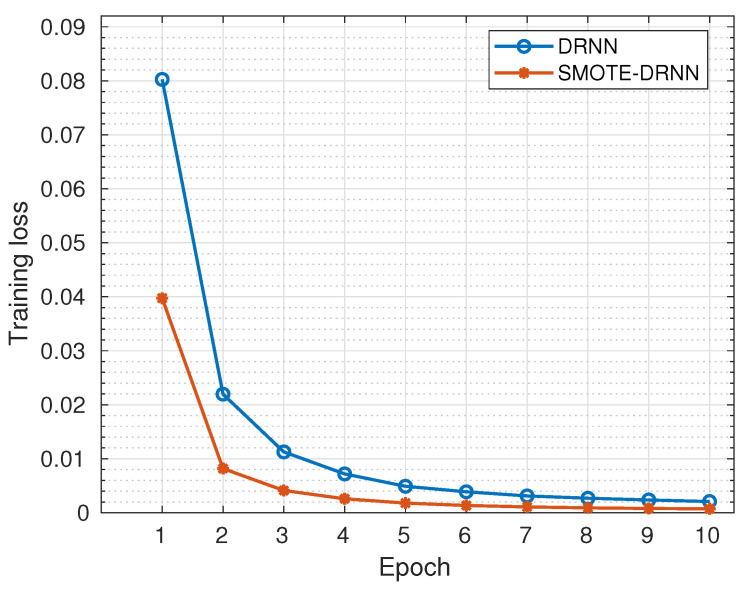
Training losses of the DRNN and SMOTE-DRNN models.

Figure 2 shows the cross-entropy losses of DRNN and SMOTE-DRNN models during training. Generally, the cross-entropy losses were reduced as the number of epochs increased from 1 to 10. Specifically, the cross-entropy loss of the DRNN model was reduced from 0.0803 to 0.0021 while that of the SMOTE-DRNN model was reduced from 0.0397 to 0.0007. At the end of model training, the cross-entropy loss of the SMOTE-DRNN model was lower by 65.14% compared to that of the DRNN model. This implies that the SMOTE-DRNN model is robust against underfitting compared to the DRNN model.

**Figure 3:**
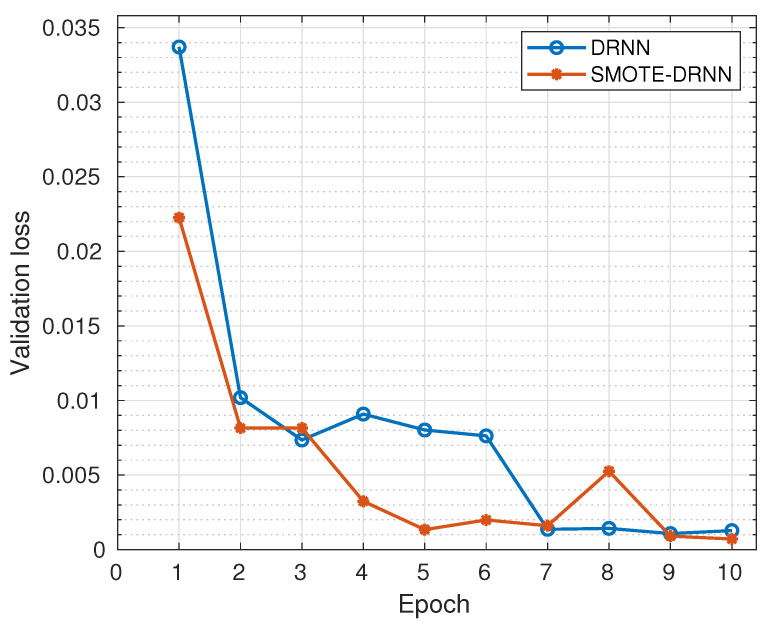
Validation losses of the DRNN and SMOTE-DRNN models.

Figure 3 shows the cross-entropy losses of the DRNN and SMOTE-DRNN models during validation. Generally, the cross-entropy losses were reduced as the number of epochs increased from 1 to 10. Specifically, the cross-entropy loss of the DRNN model was reduced from 0.0337 to 0.0013 while that of the SMOTE-DRNN model was reduced from 0.0223 to 0.0007. At the end of model training, the cross-entropy loss of the SMOTE-DRNN model was lower by 44.16% compared to that of the DRNN model. This implies that the SMOTE-DRNN model is robust against overfitting compared to the DRNN model.

The training speeds of the DRNN and SMOTE-DRNN models were evaluated based on the number of samples in the training set and the training time. The DRNN model spent 631.30 s learning the feature representation of highly imbalanced network traffic from 2,201,112 samples. On the other hand, the SMOTE-DRNN model generated more minority samples to achieve class imbalance, and this increased its computation time. The generation of minority samples took 424.09 s, and the SMOTE-DRNN model spent 1147.32 s learning the feature representation of balanced network traffic from 6,813,554 samples.

### 4.3. Classification Performance

In this subsection, we analyse the accuracy, precision, recall, F1 score, FPR, NPV, AUC, GM and MCC of the DRNN and SMOTE-DRNN models to evaluate their classification performance with network traffic samples in the test set.

Table 3 presents the accuracy, precision, recall, F1 score, FPR, NPV, AUC, GM and MCC of DRNN and SMOTE-DRNN models for each of the 11 classes. Despite the class imbalance in the training set, the accuracy and NPV of the DRNN model were very high (≈100%) and its FPR was very low (≈0%) for all of the classes. Similarly, the precision, recall, F1 score, AUC, GM and MCC of the DRNN model for the majority classes were very high (≈100%). However, the precision, recall, F1 score, AUC, GM and MCC of the DRNN model for the minority classes were relatively lower compared to those of the majority classes.

**Figure 4:**
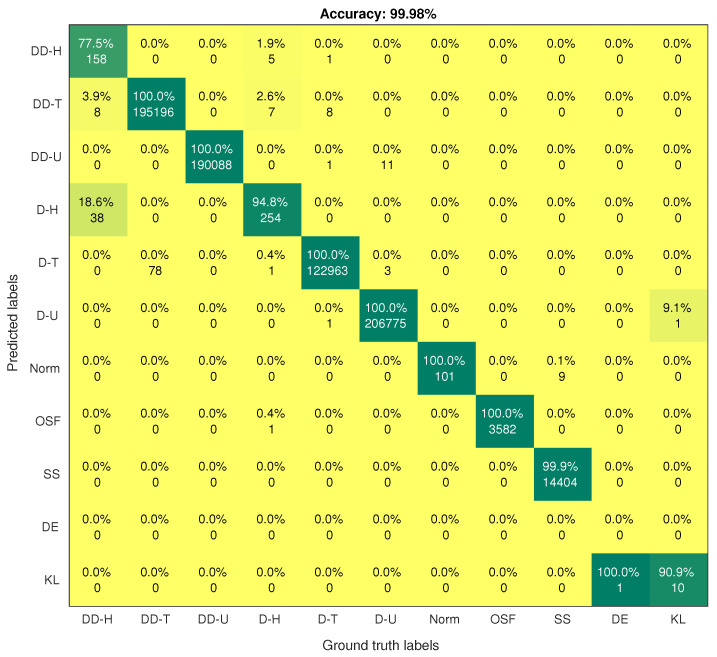
Confusion matrix of the DRNN model.

On the contrary, the SMOTE-DRNN model achieved higher precision, recall, F1 score, AUC, GM and MCC for the minority classes compared to the DRNN model. For DDH class, the precision, recall, F1 score, AUC, GM and MCC were higher by 3.66%, 21.57%, 13.64%, 10.78%, 1.85% and 13.13%, respectively. For the DH class, the precision, recall, F1 score, AUC, GM and MCC were 12.26%, 4.47%, 8.54%, 2.24%, 6.36% and 8.46%, respectively. For the Norm class, the precision, F1 score, GM and MCC were higher by 3.42%, 1.36%, 1.77% and 1.29%, respectively. Table 2 shows that there is only one sample for the DE class in the test set. Unfortunately, the DRNN model did not correctly classify this sample. The confusion matrix of the DRNN model in Figure 4 shows that TP, TN, FP and FN were 0, 733, 531, 0 and 1, respectively. Based on Equations (10)–(18), the accuracy and the NPV were 100% each; the precision, GM and MCC were undefined; while the recall and F1 score were 0% each. These seemingly improbable results were due to the high class imbalance in the training set. In this case, the DRNN model became biased in favour of the majority classes because the number of samples in these classes was relatively larger. Hence, most of the samples in the majority classes were correctly classified while the lone sample in the minority class (DE) was incorrectly classified. For the KL class, the precision, recall, F1 score and MCC were higher by 9.09% each, the AUC was higher by 4.55% while GM was higher by 4.65%. These imply that the SMOTE-DRNN model achieved better generalisation ability for minority classes.

Highly imbalanced network traffic data in the training set adversely affect the precision, recall, F1 score, AUC, GM and MCC of the DRNN model. The accuracy, FPR and NPV of DL models that were trained with highly imbalanced data can be misleading. Hence, researchers should not rely only on accuracy, FPR and NPV to evaluate DL models when the training data are highly imbalanced. Other research works that involve highly imbalanced data also confirmed that accuracy can be very high and misleading in this case [32,81].

**Figure 5:**
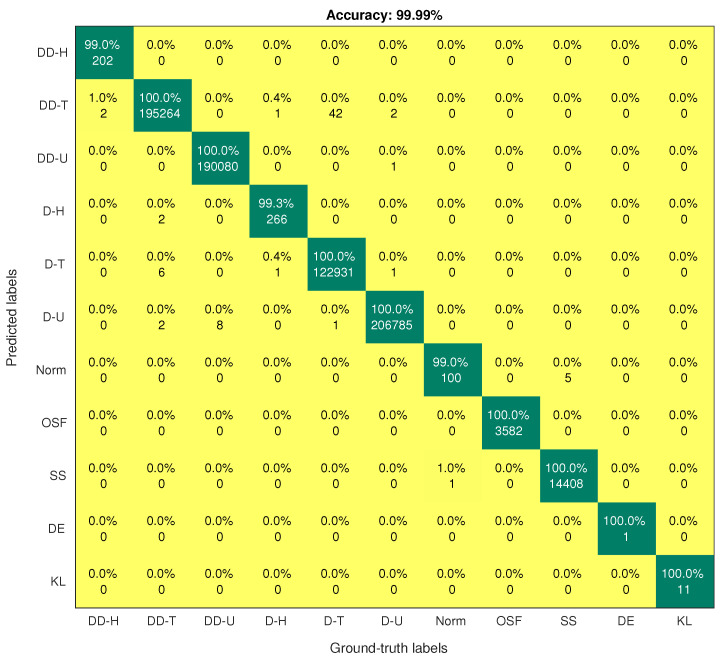
Confusion matrix of the SMOTE-DRNN model.

Figure 4 and Figure 5 show the confusion matrices of the DRNN and SMOTE-DRNN models, respectively. For DDH attack detection, the DRNN model classified 77.45% of the samples in the DDH class correctly, and the detection rate of the SMOTE-DRNN model was higher by 21.57%. For DH attack detection, the DRNN model classified 94.78% of the samples in the DH class correctly, and the detection rate of the SMOTE-DRNN model was higher by 4.48%. For DE attack detection, the DRNN model could not classify any of the samples in the DE class correctly, and the detection rate of the SMOTE-DRNN model was higher by 100%. For KL attack detection, the DRNN model classified 90.91% of the samples in KL class correctly, and the detection rate of the SMOTE-DRNN model was higher by 9.09%. These imply that the SMOTE-DRNN model is more efficient for botnet attack detection in IoT networks than the DRNN model.

The detection speeds of the DRNN and SMOTE-DRNN models were evaluated based on the number of samples in the test set and on the testing time. The DRNN model classified 733,705 samples in 3.42 s, while it took the SMOTE-DRNN model 9.81 s to classify the same number of samples.

### 4.4. Comparison with State-of-the-Art ML/DL Models

In this subsection, we compare the classification performance, the training speed and the detection speed of the SMOTE-DRNN model with those of the state-of-the-art ML/DL models. First, we analyse the accuracy, precision, recall, F1 score, FPR, NPV, AUC, GM and MCC of SMOTE-DRNN model and state-of-the-art ML/DL models to evaluate their ability to correctly detect normal traffic and the ten botnet attacks in IoT networks. Then, we evaluate the training speed based on the time taken to train the models with network traffic samples in the training set. Finally, the detection speed is evaluated based on the time taken to classify the network traffic samples in the test set.

Table 4 shows the recall of the SMOTE-DRNN model and those of the state-of-the-art ML/DL models. Unlike the state-of-the-art ML/DL models, the SMOTE-DRNN model achieved a very high recall (>99%) in each of the 11 classes. Specifically, the SMOTE-DRNN model achieved higher recall than the state-of-the-art ML/DL models in the following cases:

1. The OSF, SS and KL classes for the RNN model in [67];
2. All classes, except DU, for the SVM model in [67];
3. All classes for the RF and NB models in [67];
4. All classes, except DE, for the DL models in [68];
5. All classes, except DT, DU, DE and KL, for the RDTIDS model in [69]; and
6. The OSF, SS, DE and KL classes for the BLSTM model in [70].

**Figure 6:**
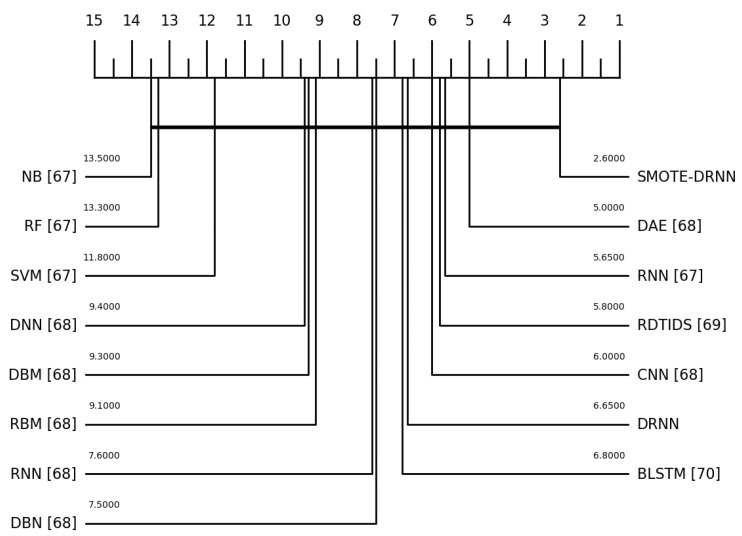
Critical distance diagram showing the mean ranks of the ML/DL models.

Recall of the state-of-the-art ML/DL models for the Norm class was not reported in the literature. Additionally, the accuracy, precision, F1 score, FPR, NPV, AUC, GM and MCC of the state-of-the-art ML/DL models in each of the 11 classes were not reported in the literature. Furthermore, we performed the Friedman test with the corresponding post hoc Nemenyi test to compare the recall of the ML and DL models. Although the differences in the recall of the models are not statistically significant, Figure 6 shows that the classification performance of the SMOTE-DRNN model is better than those of the state-of-the-art ML/DL models.

Table 5 presents the overall classification performance, training time and the testing time of the SMOTE-DRNN model and those of the state-of-the-art ML/DL models. The overall accuracy of the SMOTE-DRNN model was higher and its FPR was lower than those of the state-of-the-art ML/DL models. The overall precision, recall, F1 score, NPV, AUC, GM and MCC of the state-of-the-art ML/DL models were not reported in the literature. The time taken to train the SMOTE-DRNN model with network traffic samples in the training set was shorter than that of the state-of-the-art ML/DL models, except RNN [67], DNN [68], RDTIDS [69] and BLSTM [70]. Ferrag et al. [67] did not report the training time of the SVM, RF and NB models. The testing time of most of the state-of-the-art ML/DL models was not reported in the literature. The time taken to classify the network traffic samples in the test set was shorter than that of the RNN [67] and BLSTM [70] models.

## 5. Conclusions

In this paper, a hybrid algorithm, SMOTE-DRNN, was proposed for the multi-class classification of highly imbalanced network traffic data to detect ten classes of botnet attacks, namely DDH, DDT, DDU, DH, DT, DU, OSF, SS, DE and KL, in IoT networks. First, the SMOTE oversampling method generates more samples in the minority classes to achieve class balance. Then, DRNN learns the hierarchical feature representations from the balanced network traffic data to perform discriminative classification. The DRNN and SMOTE-DRNN models were trained, validated and tested with the Bot-IoT dataset, and their classification performance was analysed. Finally, the classification performance of the SMOTE-DRNN model was compared with the state-of-the-art ML/DL models. The major findings of this study are as follows:

1. The training and validation losses of the SMOTE-DRNN model were lower by 65.14% and 44.16%, respectively, compared to those of the DRNN model.
2. The DRNN and SMOTE-DRNN models achieved high classification performance in the majority classes (DDT, DDU, DT, DU, OSF and SS).
3. Accuracy, FPR and NPV are not suitable metrics for evaluating classification performance when the sample distribution across the classes of network traffic data is highly imbalanced.
4. For minority classes (DDH, DH, Norm, DE and KL), the precision, recall, F1 score, AUC, GM and MCC of the DRNN model were low due to high class imbalance.
5. On the other hand, the SMOTE-DRNN model achieved higher values of precision, recall, F1 score, AUC, GM and MCC in all 11 classes than the DRNN model.
6. The SMOTE-DRNN model outperformed the state-of-the-art ML/DL models.

These results showed that the SMOTE-DRNN model is robust against underfitting and overfitting. Additionally, SMOTE-DRNN demonstrated a better generalisation ability in minority classes. The major limitation/trade-off in the SMOTE-DRNN model is that it has a higher computation cost and longer training time than the DRNN model. In the future, we plan to investigate the effectiveness of the most recent versions of SMOTE (such as SMOTE-Cov) as well as generative DL models including Generative Adversarial Network (GAN) and Variational Autoencoder (VAE).

## Figures and Tables

**Table 1:** Sample distribution in the training set for the 11-class classification scenario.

Ref.	Class										
	DDH	DDT	DDU	DH	DT	DU	Norm	OSF	SS	DE	KL
[67]	594	498,602	484,127	942	317,899	526,487	4000	9002	36,700	102	106
[68]	1582	1,563,808	1,517,208	2376	985,280	1,652,759	7634	28,662	117,069	94	1175
[69]	1582	1,563,808	1,517,208	2376	985,280	1,652,759	7634	28,662	117,069	94	1175
[40]	786	781,468	759,163	1191	492,581	826,475	385	14,101	51,351	5	66
Ours	588	586,393	568,760	906	369,965	619,414	290	10,795	43,949	4	48

**Table 2:** Data samples in the training, validation and test sets.

Class	Training	Validation	Testing
DDH	588	197	204
DDT	586,393	195,713	195,274
DDU	568,760	189,407	190,088
DH	906	311	268
DT	369,965	122,861	122,974
DU	619,414	206,772	206,789
Norm	290	86	101
OSF	10,795	3537	3582
SS	43,949	14,806	14,413
DE	4	1	1
KL	48	14	11

**Table 3:** Classification performance of ML and DL models across 11 classes (all metrics are in %).

Class	Model	Accuracy	Precision	Recall	F1	FPR	NPV	AUC	GM	MCC
DDH	DRNN	99.99	96.34	77.45	85.87	0.00	99.99	88.73	98.15	86.38
	SMOTE-DRNN	100.00	100.00	99.02	99.51	0.00	100.00	99.51	100.00	99.51
DDT	DRNN	99.99	99.99	99.96	99.97	0.00	99.99	99.98	99.99	99.96
	SMOTE-DRNN	99.99	99.98	99.99	99.99	0.01	100.00	99.99	99.99	99.98
DDU	DRNN	100.00	99.99	100.00	100.00	0.00	100.00	100.00	100.00	100.00
	SMOTE-DRNN	100.00	100.00	100.00	100.00	0.00	100.00	100.00	100.00	100.00
DH	DRNN	99.99	86.99	94.78	90.71	0.01	100.00	97.39	93.27	90.79
	SMOTE-DRNN	100.00	99.25	99.25	99.25	0.00	100.00	99.63	99.63	99.25
DT	DRNN	99.99	99.93	99.99	99.96	0.01	100.00	99.99	99.97	99.95
	SMOTE-DRNN	99.99	99.99	99.97	99.98	0.00	99.99	99.98	99.99	99.98
DU	DRNN	100.00	100.00	99.99	100.00	0.00	100.00	100.00	100.00	99.99
	SMOTE-DRNN	100.00	99.99	100.00	100.00	0.00	100.00	100.00	100.00	99.99
Norm	DRNN	100.00	91.82	100.00	95.73	0.00	100.00	100.00	95.82	95.82
	SMOTE-DRNN	100.00	95.24	99.01	97.09	0.00	100.00	99.50	97.59	97.11
OSF	DRNN	100.00	99.97	100.00	99.99	0.00	100.00	100.00	99.99	99.99
	SMOTE-DRNN	100.00	100.00	100.00	100.00	0.00	100.00	100.00	100.00	100.00
SS	DRNN	100.00	100.00	99.94	99.97	0.00	100.00	99.97	100.00	99.97
	SMOTE-DRNN	100.00	99.99	99.97	99.98	0.00	100.00	99.98	100.00	99.98
DE	DRNN	100.00	N/A *	0.00	0.00	0.00	100.00	50.00	N/A *	N/A *
	SMOTE-DRNN	100.00	100.00	100.00	100.00	0.00	100.00	100.00	100.00	100.00
KL	DRNN	100.00	90.91	90.91	90.91	0.00	100.00	95.45	95.35	90.91
	SMOTE-DRNN	100.00	100.00	100.00	100.00	0.00	100.00	100.00	100.00	100.00

* ‘N/A’ implies that the metric is not applicable in this case.

**Table 4:** Recall of the ML and DL models across 11 classes (all metrics are in %).

Model	DDH	DDT	DDU	DH	DT	DU	OSF	SS	DE	KL	Rank
RNN [67]	100.00	100.00	100.00	100.00	100.00	100.00	92.22	87.91	99.75	77.91	3
SVM [67]	62.24	89.56	98.14	70.14	71.26	100.00	70.14	72.82	89.67	65.12	13
RF [67]	82.26	88.28	55.26	82.20	81.77	82.99	82.20	69.82	86.55	81.56	14
NB [67]	50.78	78.67	78.50	68.68	65.56	100	68.68	65.21	66.55	65.62	15
DNN [68]	96.62	96.22	96.12	96.70	96.63	96.53	96.14	96.43	100.00	96.76	12
RNN [68]	96.56	96.65	96.67	96.87	96.77	96.76	96.76	96.87	100.00	97.00	9
CNN [68]	97.01	97.00	97.01	97.51	97.11	97.11	97.00	97.10	100.00	98.10	5
RBM [68]	96.54	96.51	96.52	96.80	96.57	96.56	96.30	96.30	100.00	97.11	10
DBN [68]	96.72	96.60	96.62	96.91	96.72	96.83	96.61	96.60	100.00	97.66	8
DBM [68]	96.21	96.08	96.11	96.99	96.33	96.65	96.08	96.07	100.00	98.22	11
DAE [68]	97.99	97.71	97.99	98.41	98.00	98.03	97.72	97.71	100.00	98.33	2
RDTIDS [69]	93.17	95.84	98.66	77.47	100.00	100.00	98.16	99.47	100.00	100.00	4
BLSTM [70]	99.25	99.10	99.45	99.75	99.65	99.79	92.77	92.20	96.50	89.90	7
DRNN	77.45	99.96	100.00	94.78	99.99	99.99	100.00	99.94	0.00	90.91	6
SMOTE-DRNN	99.02	99.99	100.00	99.25	99.97	100.00	100.00	99.97	100.00	100.00	1

**Table 5:** Overall classification performance of the ML and DL models (all metrics are in %).

Model	Acc.	Prec.	Recall	F1 Score	FPR	NPV	AUC	GM	MCC	$T_{\mathrm{train}}$ (s)	$T_{\mathrm{test}}$ (s)
RNN [67]	99.91	-	-	-	1.28	-	-	-	-	201.70	44.23
SVM [67]	-	-	-	-	2.99	-	-	-	-	-	-
RF [67]	-	-	-	-	4.29	-	-	-	-	-	-
NB [67]	-	-	-	-	3.24	-	-	-	-	-	-
DNN [68]	98.22	-	-	-	1.14	-	-	-	-	991.60	-
RNN [68]	98.31	-	-	-	1.10	-	-	-	-	1400.60	-
CNN [68]	98.37	-	-	-	1.00	-	-	-	-	1367.20	-
RBM [68]	98.28	-	-	-	1.13	-	-	-	-	2111.90	-
DBN [68]	98.31	-	-	-	1.12	-	-	-	-	2921.70	-
DBM [68]	98.38	-	-	-	1.11	-	-	-	-	2800.10	-
DAE [68]	98.39	-	-	-	1.11	-	-	-	-	2816.20	-
RDTIDS [69]	97.00	-	-	-	1.12	-	-	-	-	195.50	2.27
BLSTM [70]	98.91	-	-	-	1.20	-	-	-	-	149.60	69.10
SMOTE-DRNN	100.00	99.50	99.75	99.62	0.00	100.00	99.87	99.74	99.62	1147.32	9.81

## Data Availability

Bot-IoT dataset is available from https://ieee-dataport.org/documents/bot-iot-dataset (accessed on 12 January 2021).
